# Fracture of the penis: an atypical presentation

**DOI:** 10.1186/1865-1380-6-32

**Published:** 2013-08-13

**Authors:** Muhammad Waseem, Ruchi Upadhyay, Ramnath Kapoor, Samuel Agyare

**Affiliations:** 1 Lincoln Medical & Mental Health Center, 234 East 149th Street, Bronx, NY 10451, USA

**Keywords:** Tunica albuginea, Corpus cavernosa, Penile shaft, Penile fracture, Injury to penis

## Abstract

**Background:**

Fracture of the penis is an uncommon injury presenting to the emergency department (ED). Personal embarrassment and social scenarios associated with this condition may result in underreporting. Patients often delay seeking medical attention, and even when they do, as in our case report, they may withhold the condition for a significant time. ED physicians need to be aware of the social inhibitions and the need for early diagnosis and prompt treatment. A delay in treatment increases the risk of complications such as ischemia, necrosis and penile deformity.

Fracture of the penis is caused by rupture of the tunica albuginea of one or both corpora cavernosa by a blunt trauma to the erect penis. Diagnosis is usually clinical as evident by the characteristic history and clinical presentation. Diagnostic modalities aid in the management of the fracture and associated injuries if present. But promptness in the recognition and initiation of treatment can significantly reduce the chances of post-injury complications.

**Findings:**

We present a case of penile fracture in a young male who presented to the ED with abdominal pain, but careful history and physical examination revealed penile fracture. A delay in diagnosis could have led to complications.

**Conclusion:**

Our case report is an attempt to emphasize the need to suspect injury to the penis in a young adult who might present to the emergency department with an entirely different complaint and also to treat any penile trauma as an emergency. This report provides evidence of an uncommon and underreported clinical entity. A review of the pertinent literature is included.

## Findings

### Introduction

Fracture of the penis is an uncommon injury. Emergency department (ED) physicians need to be aware of the urgency in the diagnosis of this condition and in the initiation of treatment as any delay increases the risk of complications. Due to the embarrassment associated with such injuries the patients may hesitate to disclose their complaint and delay seeking medical treatment. We present a case of fracture of the penile shaft in a 27-year-old male who presented to the ED with the complaint of abdominal pain for 1 week off and on. Penile fracture was diagnosed after a careful history and complete physical examination. An ultrasound confirmed the diagnosis, and the surgical repair was performed.

## Case report

A 27-year-old Hispanic male presented to the ED with abdominal pain on and off for 1 week. A careful history revealed that he also had a complaint of penile swelling. He stated that when he woke up that morning to void, he had an erection and pressed gently onto his penis to “control his erection.” He also stated that he had done this before without any adverse effects. This time, he noticed a sudden pain and gradual swelling of his penis. He denied any other trauma or voiding difficulty. There was no bleeding or hematuria, and he denied having a sexual encounter. The patient presented to the ED within an hour of the onset of symptoms.

Upon arrival to the ED, his vital signs were as follows: temperature 36.4°C (97.5°F), heart rate 67 beats per minute and blood pressure 121/72 mmHg. He was alert and oriented. The genitourinary examination revealed an uncircumcised phallus with an edematous penile foreskin extending circumferentially with ecchymosis. There was no deviation/curvature, but there was pain on compression. Palpation of the corpora revealed no significant defect. There was no blood at the urethral meatus. The remainder of the genital examination was normal. His abdominal examination was also unremarkable.

**Figure 1:**
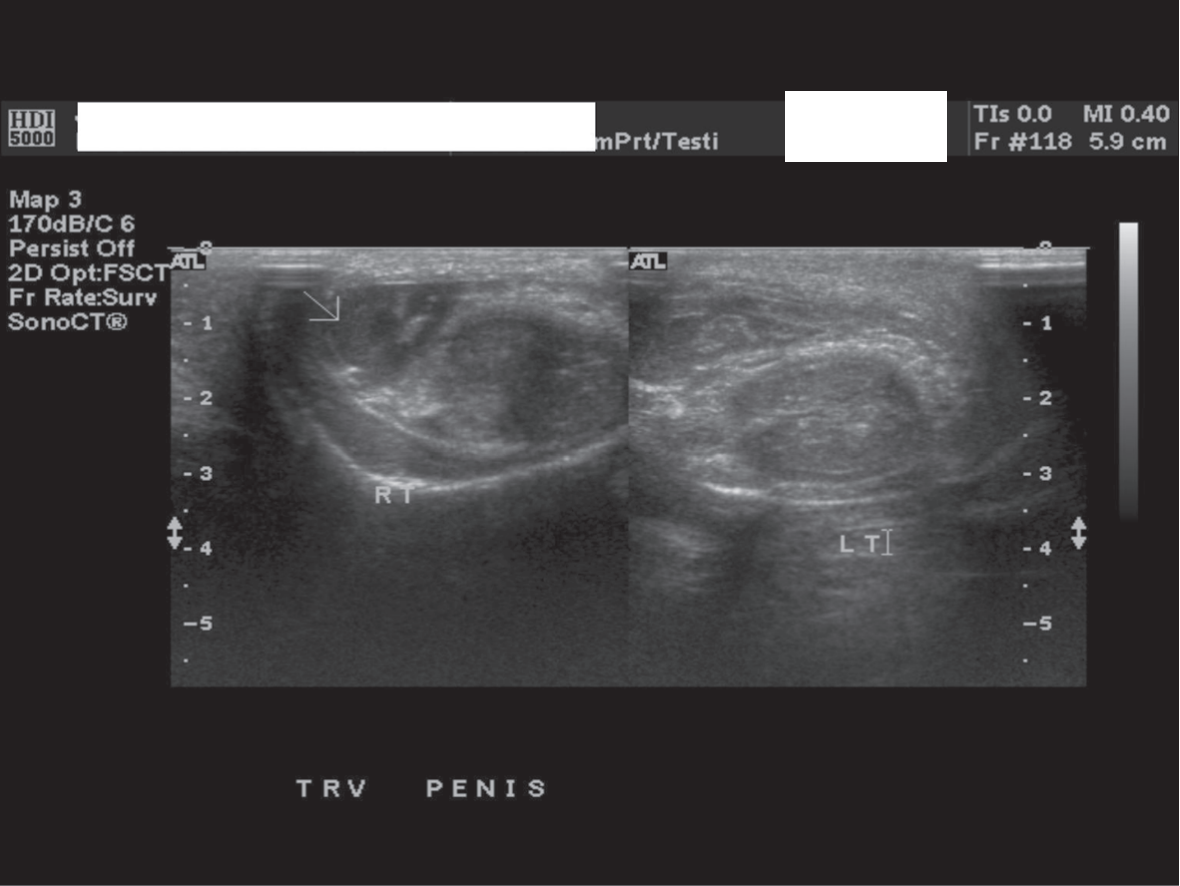
Hypoechoic mass on the lateral aspect of the right side of the penis.

**Figure 2:**
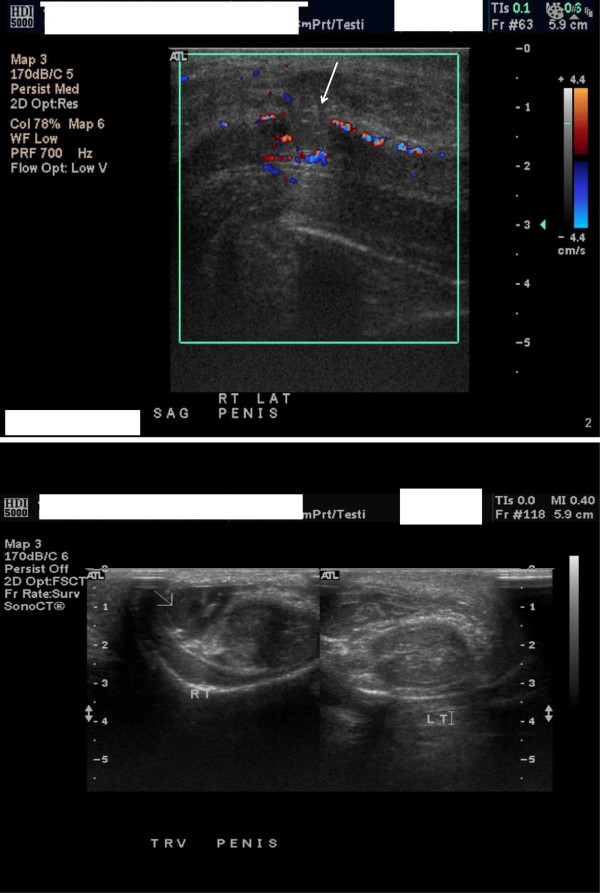
Defect in the tunica.

Based on the history and physical examination, diagnosis of a penile fracture was made. Ultrasound revealed a heterogeneous and mainly hypoechoic mass along the lateral aspect of the penis on the right side (Figure 1). There was a defect in the tunica (Figure 2) suggesting the likelihood of a fracture. The corpora cavernosa were otherwise normal. There was substantial subcutaneous edema. Surgical penile exploration was performed through a distal circumcising incision, the tear was repaired, and the hematoma was evacuated.

## Discussion

Fracture of the penis, *faux pas du coit,* is the rupture of the tunica albuginea of the corpus cavernosa. The rupture occurs when the penis is erect because the tissue of the tunica albuginea is thinner during erection and is vulnerable to a sudden increase in the intracorporeal pressure [1-3]. The urethra and corpus spongiosum may also be affected [3-7]. The tear is mostly unilateral and transverse [5,6]. Previously considered underreported, the trend of reporting cases has increased [8]. The patient’s age among the reported cases ranged from 12 to 82 years [6]. The largest numbers of cases have been reported in Mediterranean countries including Turkey [4], but a recent study noted that the number of cases reported in the Middle East and North Africa was higher than in any other countries, including the USA and Europe [6].

Among the many reported cases, the most common cause is vaginal intercourse [9,10], but blunt traumas to a tumescent shaft such as masturbation, forcibly bending the erect penis to pass urine or rolling onto an erect penis have also been reported [3,4].

The tunica albuginea is a fibroelastic sheath that encloses each corpus cavernosa, and they are surrounded by a thick fascia, Buck’s fascia. The tunica albuginea is 2 mm thick when flaccid but thins to 0.25 to 0.5 mm during erection [3,10]. An axial force to the erect penis results in a tear of tunica albuginea [11] and blood leaks out in the surrounding tissue, producing a hematoma beneath Buck’s fascia [12]. An eggplant deformity (penile swelling, discoloration and deviation) and a rolling sign (penile skin can be rolled over the clot against the affected penile shaft) [13] can be present. If the tear extends to involve Buck’s fascia, the blood leak can spread to involve the scrotum, perineum and lower abdominal wall [12]. A “butterfly sign” may be seen in the perineum if the blood leak is confined by Colles’ fascia [10].

Urethral rupture, penile vascular injury such as rupture of the penile superficial dorsal vein, deep dorsal vein, dorsal artery and deep cavernous artery are some of the associated or mimicking injuries [4,6].

### Diagnosis and evaluation

The diagnosis of penile fracture is mostly clinical [4] and easy to diagnose [5] as the history and clinical presentation are highly characteristic [7]. The patient typically presents with a sharp cracking sound in the erect penis followed by rapid detumescence [6,7]. The findings at presentation can include swelling of the penis, ecchymosis and deviation of the penis to the opposite side (as a result of hematoma and edema on the affected side) [6,7,10]. A palpable gap or defect in the penile shaft can sometimes be present [3,13]. Difficulty to pass urine or blood per urethra points to the suspicion of a urethral injury [12].

Besides careful physical examination, modalities that aid in the diagnosis are cavernography, urethrography, ultrasonography, magnetic resonance imaging, color Doppler duplex scanning and angiography [4]. Nonetheless, they are not required in most cases [14]. In certain cases, ultrasound and retrograde urethrograms should be performed to rule out associated injury and to determine surgical management [15]. If there is a suspicion of urethral involvement, retrograde urethrography should be performed and if there is an atypical presentation cavernosography and MRI can be useful [10]. In less frequent cases in which the patients do not present with classic history and examination findings, ultrasound can be useful to diagnose any breach in the tunica albuginea [16].

### Management

Any penile injury should be treated as an “emergency until proven otherwise” [17]. The management of penile fracture has changed over the years [3], and it is either conservative or surgical. Though the condition is not that common, it should be managed adeptly to avoid complications such as penile deformity or pain during intercourse. Conservative measures include splinting, cold compresses, anti-inflammatory agents, analgesia medications and anti-fibrinolytics. These are associated with significant complications such as infected hematoma, penile deformity and impotence [3,4,6,12].

Recent studies advocate immediate repair of penile fracture that have a low complication rate [3,12,18], shorter hospital stay [6] and better long-term outcome [19]. Urgent surgical repair includes procedures such as “evacuation of hematoma, ligation of bleeding vessels, debridement, suturing of tears in tunica albuginea, urethral stenting, and/or end-to-end urethral anastomosis” [3]. A circumferential subcoronal incision that exposes penile tissues is an excellent approach to expose the damaged penile tissue [6,12,19]. Other approaches include an inguinal scrotal incision [20] and longitudinal skin incision directly above the fracture site [21].

Due to the personal embarrassment and social scenarios associated with penile fracture, there can be a delay between injury and management of the conditions. Cummings et al. report that a delay of 24−48 h does not impact the postoperative functioning of the penis [11]; however, another study reports that a delay is directly related to late postoperative complications [10]. Penile deformity, pain during intercourse, erectile dysfunction, priapism, necrosis and stricture of the urethra can occur after surgical correction [22]. In our case report, the surgical repair was performed without any immediate complications.

## Conclusion

A careful history and physical examination are essential to the diagnosis of penile fracture. Patients may present to the ED with an entirely different complaint and may later reveal the actual complaint. With the social inhibitions for disclosure associated with this condition, there can be a delay in the initiation of treatment. A timely diagnosis and immediate surgical repair prevent complications and increase the chances of complete recovery.

## Abbreviations

ED: Emergency department.

## Competing interests

The authors declare that they have no competing interests.

## Authors’ contributions

MW contributed to the concept and design of the study as well as revising it for important intellectual content. RU carried out data acquisition and drafting of the manuscript. RK participated in the concept and design of the manuscript. SA gave final approval of the version to be published. All authors read and approved the final manuscript.

## References

[B1] Meares EM Traumatic rupture of the corpus cavernosum J Urol 1971 6 407 408 5549787 10.1016/s0022-5347(17)61537-4

[B2] Shaeer O Methylene blue-guided repair of fractured penis J Sex Med 2006 6 349 354 10.1111/j.1743-6109.2005.00155.x 16490031

[B3] Gamal WM Osman MM Hammady A Aldahshoury MZ Hussein MM Saleem M Penile fracture: long-term results of surgical and conservative management J Trauma 2011 6 491 493 10.1097/TA.0b013e3182093113 21278611

[B4] Eke N Fracture of penis Br J Surg 2002 6 555 565 10.1046/j.1365-2168.2002.02075.x 11972544

[B5] Asgari MA Hosseini SY Safarinejad MR Samadzadeh B Bardideh AR Penile fracture: evaluation, therapeutic approaches and long-term results J Urol 1996 6 1 148 149 10.1016/S0022-5347(01)66578-9 7490817

[B6] Atat RE Sfaxi M Benslama MR Fracture of the penis: management and long-term results of surgical treatment. Experience in 300 cases J Trauma 2008 6 121 125 10.1097/TA.0b013e31803428b3 18188109

[B7] Muentener M Suter S Hauri D Sulser T Long-term experience with surgical and conservative treatment of penile fracture J Urol 2004 6 2 576 579 10.1097/01.ju.0000131594.99785.1c 15247735

[B8] Ekwere PD Rashid MA Trends in the incidence, clinical presentation and management of traumatic rupture of the corpus cavernosum J Natl Med Assoc 2004 6 229 233 14977283 PMC2594954

[B9] Myldo H Harris CF Brown JG Blunt, penetrating and ischemic injuries to penis J Urol 2002 6 1433 1435 10.1016/S0022-5347(05)64467-9 12352411

[B10] Gottenger EE Wagner JR Penile fracture with complete urethral disruption J Trauma 2000 6 339 341 10.1097/00005373-200008000-00025 10963550

[B11] Cummings J Parra RO Boullier JA Delayed repair of penile fracture J Trauma 1998 6 1 153 154 10.1097/00005373-199807000-00032 9680030

[B12] Sawh SL O’Leary MP Ferreira MD Berry AM Maharaj D Fractured penis: a review Int J Impot Res 2008 6 4 366 369 10.1038/ijir.2008.12 18418392

[B13] Zargooshi J Penile fracture in Kermanshah, Iran: report of 172 cases J Urol 2000 6 364 366 10.1016/S0022-5347(05)67361-2 10893586

[B14] Armenakas NA Hochberg DA Fracchia JA Traumatic avulsion of the dorsal penile artery mimicking a penile fracture J Urol 2001 6 619 10.1016/S0022-5347(05)66005-3 11458089

[B15] Hawkins D Jones JS Bush C Penile fracture: evaluation and management: 85 Ann Emerg Med 2009 6 (3) Sup 1 S28

[B16] Nomura JT Sierzenski PR Ultrasound diagnosis of penile fracture J Emerg Med 2010 6 362 365 10.1016/j.jemermed.2008.03.010 18835513

[B17] Dubin J Davis JE Penile emergencies Emerg Med Clin North Am 2011 6 485 499 10.1016/j.emc.2011.04.006 21782070

[B18] Akgul T Ayyildiz A Cebeci O Effect of cyanoacrylic glue on penile fracture: an experimental study J Urol 2008 6 2 749 752 10.1016/j.juro.2008.03.181 18554635

[B19] Patel A Kotkin L Isolated urethral injury after coitus-related penile trauma J Trauma 2010 6 4 E89 E90 10.1097/TA.0b013e31818d0e2d 20386267

[B20] Seftel AD Haas CA Vafa A Brown SL Inguinal scrotal incision for penile fracture J Urol 1998 6 182 184 10.1016/S0022-5347(01)64051-5 9400467

[B21] Naraynsingh V Maharaj D Kuruvilla T Ramsewak R Simple repair of fractured penis J R Coll Surg Edinb 1998 6 97 98 9621531

[B22] Minns AB Yafai S Penile fracture in a patient presenting with groin pain J Emerg Med 2011 6 441 442 10.1016/j.jemermed.2008.08.005 19128917

